# Utilization of preconception care and associated factors among reproductive age group women in Debre Birhan town, North Shewa, Ethiopia

**DOI:** 10.1186/s12978-019-0758-x

**Published:** 2019-07-05

**Authors:** Tesfanesh Lemma Demisse, Samuel Abdu Aliyu, Sena Belina Kitila, Tiwabwork Tekalign Tafesse, Kelemu Abebe Gelaw, Mulualem Silesh Zerihun

**Affiliations:** 1 Department of Midwifery, College of Health Science and Medicine, Wolaita Sodo University, Wolaita Sodo, Ethiopia; 2 School of Nursing and Midwifery, Institute of Health, Faculty of health sciences, Jimma University, Jimma, Ethiopia; 3 Department of Nursing, College of Health Science and Medicine, Wolaita Sodo University, Wolaita Sodo, Ethiopia; 4 Department of Midwifery, College of Health Science and Medicine, Debre Birhan University, Debre Birhan, Ethiopia

**Keywords:** Preconception care, Utilization, Reproductive age group women, Debre Birhan town, Ethiopia

## Abstract

**Background:**

Preconception care refers to things women can do before and between pregnancies to increase the chance of having a healthy baby and being a healthy mother. Unfortunately, millions of women in the world do not have access to pre-pregnancy, pregnancy health services and childbirth with suitable quality. Therefore, addressing this significant gap and coming up with the necessary information is helpful to improve maternal and child health in our country. So, this study was aimed to assess the utilization of preconception care and associated factors among reproductive age group women in Debre Birhan Town, North Shewa, Ethiopia.

**Methods:**

A mixed method of community based cross-sectional study was employed from March 1st to 30; 2017. Systematic sampling technique was used to select a total of 424 reproductive age women. The data were collected using pre-tested and structured questionnaire and eight in-depth interviews were done using an interview guide. The collected data were coded and entered into Epi data 3.5.1 and exported to SPSS version 21 for cleaning and analysis. Logistic regression was run to look for the association between dependent and explanatory variables; and using variables which have *p*-value ≤0.25 binary logistic regression was fitted. Association presented in Odds ratio with 95% confidence interval and significance determined at *P*-value less than 0.05.

**Result:**

A total of 410 subjects were participated with a response rate of 96.7%. The overall utilization of Preconception care was 13.4%. Woman’s age, marital status, knowledge and availability of unit for preconception care were significantly associated with utilization of preconception care with (AOR: 3.567; 95% CI: 1.082, 11.758), (AOR: 0.062; 95% CI: 0.007, 0.585), (AOR = 6.263; 95% CI: 2.855, 13.739) and AOR: 13.938; 95% CI: 3.516, 55.251) respectively.

**Conclusions:**

The finding of this study showed that women’s utilization of preconception care is relatively low. A woman’s age, marital status, educational status, knowledge about preconception care services and availability of unit for preconception care were factors affecting utilization of preconception care. Therefore, establishing preconception care strategies which can address all the components of the care will be essential when designing effective implementation strategies for improving the uptake of preconception care.

## Plain English summary

Preconception care is any intervention provided to women and couples of reproductive age, regardless of pregnancy status or desire, before pregnancy, to improve health outcomes for women, newborns and children.

In this study, respondents were asked via close ended structured questionnaire through face to face interviews whether or not they utilize preconception health care services before they become pregnant.

A total of 410 subjects were participated with a response rate of 96.7%. Of this, majority of the participants 304 (74.1%) were married and 118 (28.8%) of women were government employers. And eight key informants also asked for an in-depth interview to identify health facility related factors.

The study revealed that overall utilization of preconception care by reproductive age group women was 13.4%. A woman’s age, marital status, educational status, knowledge about preconception care services and availability of unit for preconception care were found to be the factors associated with preconception care utilization.

S of preconception care is relatively low. Therefore, establishing preconception care strategies which can address all the components of the care will be essential when designing effective implementation strategies for improving the uptake of preconception care.

## Background

Preconception care is defined as a set of interventions and/or programmes that aims to identify and enable informed decision-making to modify biomedical, behavioral, and (psycho-) social risks to parental health and the health of their future child, through counseling, prevention and management, emphasizing those factors that must be acted on before conception and in early pregnancy, to have maximal impact and/or choice [1].

In 2012, The WHO has organized a meeting to develop a global consensus on preconception care to reduce maternal and childhood mortality and morbidity. According to the review, lists of programmes included in preconceptions are Tobacco use prevention and cessation, Nutrition, Vaccine, Fertility and infertility, Female genital mutilation, HIV testing and counseling, Mental health, Substance use, Intimate partner and sexual violence, Premarital counseling, Genetic counseling, Maternal and child health, Adolescent-friendly services, and Occupational health [2].

Even if maternal health has significantly improved in the twenty-first century, but too many women continue to die or suffer severe pregnancy complications every year [3]. Worldwide by the end of 2015, 3 03000 women will have died during and following pregnancy and childbirth [4]. In the same year, an estimated 5.9 million children under 5 years of age died, of those deaths, 45% were newborns [5] and Preterm birth complications are the leading cause which is responsible for nearly 1 million deaths in 2015 [6]. This risk of maternal and infant mortality and pregnancy-related complications can be reduced by increasing access to quality preconception (before pregnancy) and interconception (between pregnancies) care [7].

Sustainable Development Goal (SDG) number three puts a target to reduce the global maternal mortality ratio to less than 70 per 100,000 live births and newborn mortality at least as low as 12 per 1000 live births by the year 2030 [8]. Preconception care has a positive impact on reduction in mortality and decrease the risk of adverse health effects for the woman, fetus, and neonate by optimizing the woman’s health and knowledge before planning and conceiving a pregnancy [9].

The World Health Organization (WHO) recently stated that globally four out of 10 women report that their pregnancies were unplanned. As a result, 40% of pregnancies miss the essential health interventions required prior to pregnancy [10]. The Center for Disease Control (CDC) recommends risk assessment and counseling for all women of childbearing age as part of primary health care visits in order to improve pregnancy outcomes [7]. But unfortunately, millions of women in the world do not have access to pre-pregnancy, pregnancy health services and childbirth with suitable quality, especially poor, illiterate women or those in deprived areas [11]. According to Pregnancy Risk Assessment Monitoring System (PRAMS) report 2004–2008 in Utah, only 32% of the 30,481 reproductive aged individuals reported they had received preconception counseling, with significantly low rates among those with unintended pregnancy [12].

In low-income countries, preconception care has not been widely implemented because its aims and objectives are not widely understood and accepted [13]. The SDG report 2014 indicates that since 1990 to 2015 maternal mortalities has reduced by only 50% and the proportion of mothers who do not survive childbirth compared to those who survive childbirth in the developing regions is 14 times higher than in developed nations [14]. A negative outcome of an expectant mother directly affects the fetus or the newborn [15]. So preconception care has found a place in the continuum of care aimed at improving maternal, newborn and child health in low and middle-income countries [16].

Preconception care utilization in developing countries like Sudan, Brazil and Sri Lanka is 9% [17], 15.9% [18] and 27.2% [19] respectively. While utilization of preconception care is slightly higher in developed countries like China, London, Saudi Arabia, and Maryland which is 20.6% [20], 27% [21], 29.3% [22] and 32% [23] respectively. According to findings from different articles utilization of preconception care is affected by age, educational status, ethnicity, employment status, marital status, history of family planning use, having a previous miscarriage, stillbirth or termination due to fetal abnormality, pregnancy intention, parity, gravidity, knowledge of preconception care, availability and accessibility of the services. [12, 19–21, 23–25]

Despite its importance in promoting maternal health, contributing to a healthy pregnancy, little is known about how Ethiopian women, especially reproductive age women have been preparing for pregnancy and what they know about preconception preparation. So this study was designed to assess the preconception care utilization and determine factors that influence the uptake and utilization of preconception care among reproductive age group women.

## Methods

### Study design and settings

A community based cross-sectional study design was conducted in Debre Birhan Town, North Shoa, Ethiopia; from March 1st to March 30, 2017. The town is located 130 km northeast of Addis Ababa. The town is divided into 9 kebeles that has a total area of 142.71 km with an average elevation of 2840 m above sea level. According to the information obtained from the district health office, in 2015/16, the total population size of the district is put as 92,887 out of which 54.78% (50,883) are women. From those women, 23.58% (21,903) are age between 15 and 49. There are one referral hospital, four health centers, one university and four colleges under the government and one private hospital and 17 private clinics in the town.

### Study participants

All reproductive age women who lived in Debre Birhan town were the source and study population. All reproductive age group women who had a history of pregnancy and lived in Debre Birhan Town for 6 months and above were included under the study.

### Sampling technique and procedure

A sample size of 424 was determined by using a single population proportion formula with the following assumptions: Since there is no local data for the value of p, the prevalence of 50% is taken. D is the expected margin of error (5%), Z, the standard score corresponding to a 95% confidence interval and α, the risk of rejecting the null hypothesis (0.05) and 10% non-response rate.

The entire nine kebeles of Debre Birhan town was taken. A total number of households in each kebeles were taken from the 2017 work plan of the district health office. The sample size for each kebeles was determined proportionally to the number of households within each kebeles. To reach the study unit systematic sampling technique was used in the kebeles. The sampling interval of the households in each kebeles was determined by dividing the total number of households in the specific Kebele to the allocated sample size (N/n) th which is forty- two. The first house was selected randomly in one place and every 42nd house for all kebeles was asked. When there was no eligible woman in the selected house, a nearby house was asked. In case of more than one eligible woman were encountered in the selected household, a lottery method was used to determine which woman would be interviewed.

In the qualitative study, eight key informants seven from health institution and one from Woreda health office were selected for in-depth interview using purposive sampling technique. The purpose was health professionals that were working on maternal, sexual and reproductive health services and relating issues.

### Study variable

The dependent variable is the utilization of preconception care and the independent variables were Socio-demographic characteristics, obstetric and gynecologic history, Knowledge of preconception care and health service related factors.

### Operational definitions

Preconception care: Any interventions either advice or treatment, and lifestyle modification women received regarding components of preconception care before being pregnant [26].(Preconception care components in this study is HIV testing and counseling, STI screening and treatment, Infertility/sub-fertility treatment, Nutrition, Ferrous supplementation, Immunization, Advice on cessation of alcohol, Advice on cessation of cigarette smoking).

Unit for preconception care: is a unit or room where women’s received preconception care before being pregnant.

Preconception care utilization: If women received any interventions either advice or treatment, and lifestyle modification regarding components of preconception care at least once before being pregnant.

Good knowledge: Those who have scored above or equal to 50% of correct responses to preconception care knowledge questions [26].

Poor knowledge: Those who have scored less than 50% of correct responses to preconception care knowledge questions [26].

### Data collection instrument/process

Data was collected using a pre-tested structured questionnaire through face to face interview. The study questionnaire consists of different parts for data collection up on the tool adapted from previous literature in different parts of the world and modified according to the local context. Six (6) Diploma Nurse and three Bsc Midwife supervisors who were familiar with the study area and experienced in data collection were hired to collect the data after attending 1 day training on the aim of the study, content, objective, data collection and interviewing technique and issue on confidentiality. During the data collection, regular supportive supervision and discussion with data collectors and supervisors were done. Every day, the supervisors have checked all the filled questionnaires for completion and clarity.

A semi-structured in-depth interview guide was used to collect the qualitative data. The principal investigator has collected the data through the assistance of one Msc degree who are experienced in qualitative data collection.

### Data analysis

The collected data were first checked manually for completeness, missed values, unlikely responses and then coded, entered using Epi data version 3.5.1. Then cleaned and analyzed using SPSS version 20. Descriptive statistics were computed to determine frequencies and summary statistics (mean, standard deviation, and percentage) to describe the study population in relation to socio-demographic and other relevant variables. Data were presented using tables, graphs, and figures. Variables with a *P* value < 0.25 in bivariate analysis were transferred to multivariate analysis. Multiple logistic regressions were done to test the presence of an association between predictors and dependent variables. *P* value ≤0.05, at 95% confidence interval was considered as a cut point to declare the presence of statistically significant association. The odds ratio was used to determine the direction and strength of the association.

For the qualitative part, thematic analyses were employed to extract meanings out of the texts manually. First, the data was transcribed and coded. Then categorized and thematized in line with Pre-determined thematic areas. Factors affecting utilization of preconception care as explained by the participants were thematically categorized to knowledge and health facility related factors. Then finally results were presented by supporting with the quantitative data.

### Data quality control

The data collection tool was translated into local language, Amharic by experts in both languages and was translated back to English by another person to ensure consistency and accuracy. Training was given to both the data collectors and supervisors for 1 day on the purpose of the study, data collection tools, and procedure, how to interview, handling ethical issues and maintaining confidentiality and privacy. Each supervisor and Principal investigator was supervised data collectors and checked all the filled questionnaires for completion, clarity, and consistency on daily bases. The questionnaire was pre-tested on 5% of the calculated sample size to familiarize enumerators with the administration of the interview process and for ensuring consistency. The pre-test study covered 22 eligible reproductive age group women who are living in Shewarobit town, which become out of the main study town 2 weeks before the commencement of the main data collection. Debriefing sessions were held with the pre-test field staff and the questionnaires were modified based on lessons drawn from the pre-test. The validity of the tool was also approved by experts.

### Ethical statement

Ethical clearance and approval letter to conduct the study were obtained from Jimma University institutional review board and a letter of cooperation was taken from Jimma University institute of health to Debre Birhan town health bureau. Written consent was obtained from the study participants after explaining the study objectives and procedures. The right to refuse not to participate in the study any time they want was assured and Confidentiality of the information was ensured by coding. The interview was undertaken privately in a separate area. Only authorized person was getting access to the raw data collected from the field.

## Result

### Participant characteristics

A total of 410 reproductive age women were participated with a response rate of 96.7%. The mean age of the participants was 28.8 years, with a standard deviation of + 6.739 and with a maximum and minimum age of 46 and 18 years respectively. Three hundred eight (75.1%) of the participants were Amhara and 310 (75.6%) were Orthodox Christian. One hundred and sixty seven (40.7%) of respondents had a monthly household income of 40–120 Dollar and 141 (34.4%) were educational level of more than secondary school. The majority of the participants 304 (74.1%) was married and 118 (28.8%) of women were government employers. One hundred thirty four (43.4%) and 150 (49.3%) of the participant’s husband were government employees and more than secondary school respectively. The majority of the participants 208 (50.7%) has a family size below the mean 4 (See Table 1).

**Table 1:** Distribution of study subjects by socio-demographic characteristics in Debre Birhan town, North Shewa, Ethiopia, March 2017(*n* = 410)

Characteristics	Category	Frequency (N)	Percent (%)
Age of mother	15–24	114	27.8
	25–34	207	50.5
	35–49	89	21.7
Religion	Orthodox	310	75.6
	Muslim	45	11
	Protestant	41	10
	Catholic	14	3.4
Ethnicity	Amhara	308	75.1
	Oromo	60	14.6
	Tigray	23	5.6
	Guragie	19	4.6
Marital status	Married	304	74.1
	Single	78	19.0
	Others^a^	28	6.8
Educational status of women	No formal education	60	14.6
	Primary school	73	17.8
	Secondary school	136	33.2
	More than secondary	141	34.4
Women occupation	House wife	103	25.1
	Government employee	118	28.8
	Market trade vendor	90	22.0
	Student	73	17.8
	Daily laborer	26	6.3
Husband education (*N* = 304)	No formal education	21	6.9
	Primary school	53	17.4
	Secondary school	80	26.3
	More than secondary	150	49.3
Husband occupation (*n* = 304)	Government employee	132	43.4
	Market trade vendor	106	34.9
	Daily laborer	48	15.8
	Others^b^	18	5.9
Total household income per month in ETB	< 40 Dollar	70	17.1
	40–120 Dollar	167	40.7
	121–200 Dollar	109	26.6
	> 200 Dollar	64	15.6
Family size	< 4	208	50.7
	> = 4	202	49.3

Others^a^Widowed and Divorced

Others^b^farmer and student

More than half 213 (52.0%) of the respondents were primigravida and 216 (54.1%) of them were Primiparous. The majority 275 (67.1%) of respondents had a history of family planning use. Twenty three (5.6%) of the respondents had a history of spontaneous abortion. Thirteen (3.2%), 11(2.7%), 10(2.4%) and 9(2.2%) of the respondents had history of still birth, preterm birth, congenital abnormality and neonatal death respectively.

### Preconception care knowledge score

**Fig. 1:**
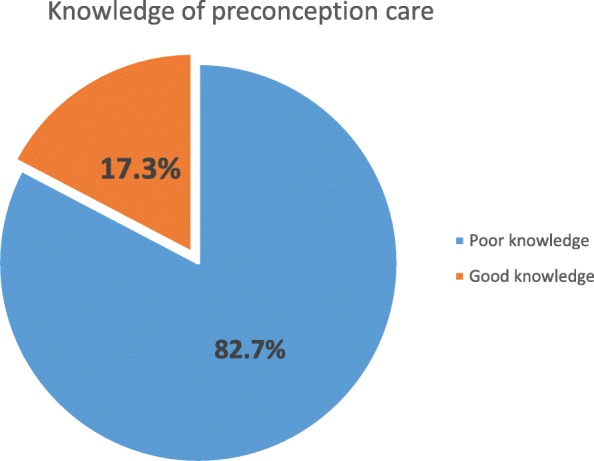
Women’s general Knowledge about preconception care in Debre Birhan Town, North Shewa, Ethiopia, March 2017 (*n* = 410). Red color -women who had good knowledge, Blue color-women who had poor knowledge

Among the total of 410 participants, 145 (35.4%) of women have heard about preconception care before. For those who have heard about preconception care; the major source of information was health workers 92 (63%) and minority 39 (26.9%) of them have heard from friends/family. Women’s knowledge on preconception care were measured based on correct response using six preconception care knowledge questions and the question was scored out of 18 points. Women’s knowledge was categorized by using 50% as a cutoff point. The minimum and maximum score of participants was 0 and 18 respectively. Seventy one (17.3%) of them had good knowledge on preconception care (Fig. 1).

### Health facility related factors

Availability of a health facility in the study area was also assessed. Accordingly, all 410 (100%) of respondents confirm the availability of health facility (hospital/health center). Majority 307 (74.9%) and 287 (70%) of the participants confirm that there were availability of adequate medication and laboratory service respectively. Only 23 (5.6%) of the participants confirm that there were unit for delivery of preconception care. Two hundred and fifty (61%) of participants mentioned that time to reach the nearby health facility on foot took 5 km (see Table 2).

**Table 2:** Availability and accessibility of health facility in Debre Birhan Town, North Shewa, Ethiopia, March 2017

Variables	Frequency (N)	Percent (%)
Availability of adequate laboratory service		
Yes	287	70
No	32	7.8
Don’t know	91	22.2
Availability of adequate medication		
Yes	307	74.9
No	31	7.6
Don’t know	72	17.6
Availability of unit for preconception care		
Yes	23	5.6%
No	135	32.9%
Don’t know	252	61.5
Time to reach health facility (on foot)		
< 5 km	250	61.0
≥ 5 km	160	39.0

### Utilization of preconception care

**Fig. 2:**
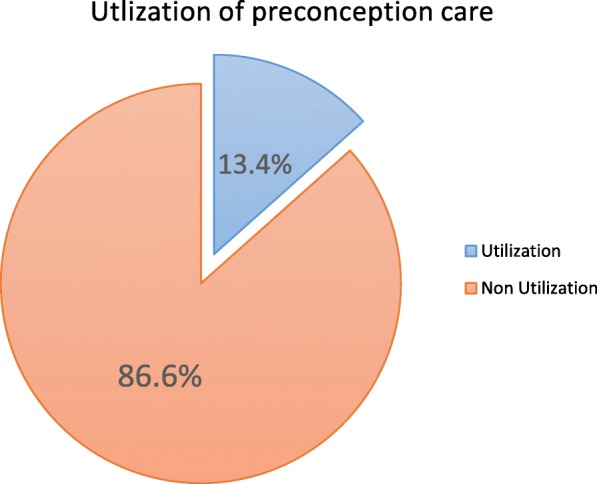
Overall utilization of preconception care among reproductive age group women in Debre Birhan Town, North Shewa, Ethiopia, March 2017 (*n* = 410). Blue color - women who utilize preconception care, Red color -women who do not utilize preconception care

Fifty five (13.4%) women’s was utilized preconception care services (Fig. 2). Among those HIV testing and counseling was majorly utilized 51 (92.7%) service. Fourteen (25.5%) of the participants who received of preconception care faced challenges during care. Among the participants who faced challenges majority 10 (71.4%) told consumption of extended time during care provision and negligence from health care providers. Among the study participants who are married majority 48 (92.3%) had support from their husbands for preconception screening. Only 4 (7.7%) of the participants have no support towards the care from their husband, among those all of the husbands due to lack of knowledge on how preconception care benefits the couples.

### Factors associated with utilization of preconception care

There were thirteen variables in binary logistic regression which had *p* value of ≤0.25; and became candidate for multiple logistic regression; women’s age, education and occupation, marital status, Total household income per month, family size, knowledge of women about preconception care services, history of preterm birth, history of contraceptive use, Preexisting chronic medical problem, availability of adequate laboratory service, availability of adequate medication, availability of unit for preconception care. In the multiple logistic analysis; women’s age, marital status, educational status, knowledge about preconception care services and availability of unit for preconception care were significantly associated (*p* < 0.05) (See Table 3).

**Table 3:** Factors associated with utilization of preconception care among reproductive age group women in Debre Birhan Town, North Shewa, Ethiopia, March 2017 (*n* = 410)

	Variable	Utilization of PCC		COR,95%CI	AOR,95%CI
		Yes	No		
Age of the women	15–24	8(14.5%)	106(29.9%)		1
	25–34	32(58.2%)	175(49.3%)	2.423(1.076,5.454)	1.373 (0.475, 3.972)
	35–49	15(27.3%)	74(20.8%)	2.686(1.083,6.659)	3.567 (1.082, 11.758)
Marital status of women	Married	52(94.5%)	252(71.0%)		1
	Single	1(1.8%)	77(21.7%)	0.063(0.009,0.463)	0.062 (0.007, 0.585)
	Others^1^	2(3.6%)	26(7.3%)	0.373(0.086,1.619)	1.394 (0.269, 7.234)
Educational status of women	No formal education	1(1.8%)	59(16.6%)	0.055(0.007, 0.416)	0.076 (0.009, 0.639)
	Primary school	6(10.9%)	67(18.9%)	0.293(0.117,0.737)	0.380(0.124, 1.160)
	Secondary school	15(27.3%)	121(34.1%)	0.406(0.209, 0.787)	0.497(0.221, 1.119)
	More than secondary	33(60.0%)	108(30.4%)		1
Occupation of women	House wife	12(21.8%)	91(25.6%)		1
	Government employee	30(54.5%)	88(24.8%)	2.585(0.245, 5.369)	0.562(0.180,1.753)
	Market trade vendor	11(20.0%)	79(22.3%)	1.056(0.442,2.525)	0.719(0.233,2.220)
	Student	1(1.8%)	72(20.3%)	0.303(0.038,2.446)	0.650(0.064,6.604)
	Daily laborer	1(1.8%)	25(7.0%)	0.105(0.013, 0.829)	0.242(0.015,3.777)
Total house hold income per month	< 40 dollar	1(1.8%)	69(19.4%)	0.030(0.004,0.229)	0.119 (0.011,1.324)
	40–120 dollar	13(23.6%)	154(43.4%)	0.173(0.080,0.373)	0.441(0.148,1.316)
	121–200 dollar	20(5.9%)	89(25.1%)	0.460(0.226,0.938)	1.658(0.260,1.666)
	> 200 dollar	21(5.9%)	43(12.1%)		1
Family size	< 4	23(41.8%)	185(52.1%)		1
	≥4	32(58.2%)	170(47.9%)	1.514(0.852,2.690)	1.101(0.450,2.692)
History of preterm birth	Yes	3(5.5%)	8(2.3%)	2.502(0.643,9.736)	3.824(0.659,22.201)
	No	52(94.5%)	347(97.7%)		1
History of contraceptive use	Yes	45(81.8%)	230(64.8%)	2.446 (1.192,5.019)	1.008(0.318,3.156)
	No	10(18.2%)	125(35.8%)		1
preexisting medical condition	Yes	10(18.2%)	40(11.3%)	1.750(0.818,3.742)	1.999(0.786,5.087)
	No	45(81.8%)	315(88.7%)		1
Knowledge of women on PCC	Poor knowledge	28(50.9%)	331(87.6%)		1
	Good knowledge	27(49.1%)	44(12.4%)	6.816(3.682,12.616)**	6.263(2.855,13.739)
Availability of adequate laboratory service	Yes	43(78.2%)	244(68.7%)		1
	No	6(10.9%)	26(7.3%)	1.309(0.509,3.369)	1.033(0.271,3.933)
	Don’t know	6(10.9%)	85(23.9%)	0.401(0.165,0.975)	0.735(0.162,3.327)
Availability of adequate medication	Yes	48(87.3%)	259(73.0%)		1
	No	3(5.5%)	28(7.9%)	0.578(169, 1.978)	0.416(0.088,1.969)
	Don’t know	4(7.3%)	68(19.2%)	0..317(111, 0.911)	0.661(0.177,2.475)
Availability of unit for PPC	Yes	6(10.9%)	17(4.8%)	8.541(2.772,26.316)**	13.938(3.516,55.251)
	No	39(70.9%)	96(27.0%)	9.831(4.720,20.476)**	10.027(4.331,23.320)
	Don’t know	10(18.2%)	242(68.2%)		1

** *P*- < 0.05 statically significant

Women whose age is 34–49 years were 3.6 times more likely to utilize preconception care than women whose age is 15–24 years (AOR: 3.567; 95% CI: 1.082, 11.758). Accordingly, women who have good knowledge of preconception care services were 6.2 times more likely to utilize preconception care than that of poor knowledge (AOR = 6.263; 95% CI: 2.855, 13.739).

The Qualitative study also supports this finding; lack of awareness about preconception care is the major problem that affects the utilization of the service among reproductive age groups.

As one of a 30 year old female participant said: *“… the main problem here is lack of familiarity and knowledge of PCC both among women’s as well as healthcare professionals’, for example, we provide preconception information when we directly asked by the women rather than spontaneously offers it.”*

Similarly a 34 year old male participant said: *“there is not a concept of preconception in our society, so using special way of giving information is very important, especially media have a great role on this.”*

In addition, as explained by a 28 year old female participant: *“Our problem is that our target group does not have information on pregnancy and health and has not yet believed that what the benefits of PCC, so they do not take the service.”*

In addition Women who mentioned there is an available unit for preconception care were 14 times more likely to utilize preconception care than women who don’t know the availability of unit for preconception care (AOR: 13.938;95% CI: 3.516,55.251). Also, women who mentioned that there is no available unit for preconception care delivery were 10 times more likely to utilize preconception care than women who don’t know the availability of unit for preconception care (AOR: 10.027;95% CI: 4.331,23.320).

Most of the participants of in-depth interview also stated that, the lack of a centrally coordinated and comprehensive offer of PCC was also another issue that was raised as an important reason for the low uptake of PCC amongst reproductive age groups.

As explained by a 33 year old male participant said: *“even though the government believes in this program, there is Poor organization and coordination of PCC during routine care. Due to that there is limited information about the availability of the service among women’s regarding PCC. so many women’s comes to health institution when she gets pregnant.”*

In addition, another a 32 year old female participant stated that *“Eventhouh there is no available unit for delivery of PCC alone in our health care setting, PCC is given by integration with other maternal and reproductive health care services like ANC and family planning services.”*

However, women who did not attend formal education were 92.4% (AOR: 0.076; 95% CI: 0.009, 0.639) less likely to utilize preconception care than women whose educational level more than secondary. Also single women were 93.8% (AOR: 0.062; 95% CI: 0.007, 0.585) less likely to utilize preconception care than married.

## Discussion

The overall utilization of preconception care by reproductive age group women in this study was 13.4%, which is higher than a study carried out in Sudan (9%) [17]. This might be due to in Sudan, the study is done only on small sample size of reproductive age women’s with Rheumatic heart disease but in this study utilization of preconception care was assessed by community based study design with maximum representative sample size which make the study comprehensive. However, it is significantly lower than the finding from Brazil (15.9) [18] and Sri Lanka (27.2%) [19]. The possible explanation might be due to difference in the study setting, study participants and health care system of the countries.

This finding also lower than study conducted in China (20.6%) [20] and London (27%) [21]**.** This might be due to the fact that there were difference in culture, health care system and educational status of women. It is also significantly lower than study conducted in Saudi Arabia (29.3%) [22] and Maryland (32%) [23]. This might be due to the fact that in Maryland it is a cumulative result of different studies that were conducted on different study participants with large sample size in different study settings and period.

It was observed that women whose age is from 34 to 49 years were 3.6 times more likely to utilize preconception care than women whose age is 15-24 years (AOR: 3.567; 95% CI: 1.082,11.758). This finding is not consistent with studies conducted in Utah [12], Brazil [18] and China [20]. Older women may have thought they were not at an appropriate age for conception and they are at risk for pregnancy complication. Thus, they tended to use Preconception care.

This study also indicated that significant association was noted between women’s knowledge and PCC utilization. Women’s who have good knowledge of preconception care services were 6.2 times more likely to utilize preconception care than that of poor knowledge (AOR: 6.263; 95% CI: 2.855, 13.739). This is in line with a finding from china [20], Saudi Arabia [22] and Nigeria [24]. This might be due to an in-depth knowledge of preconception care may increase women’s understanding and awareness of the purpose and importance of PCC, and thus, their use of this service.

As explained in the qualitative part of this study Most of the participants also agree with the quantitative finding giving information and education regarding preconception care is essential to increase knowledge and utilization of the care.

However, women who did not attend formal education were 92.4% (AOR: 0.076; 95% CI: 0.009, 0.639) less likely to utilize preconception care than women whose educational level more than secondary. The finding of this study is consistent with a study done in Utah [12], Sri Lanka [19], London [21] and Oklahoma [25]. This might be due to the fact that the women’s with lower educational level might be less exposed to information regarding to preconception care. The information gap might an able them to understand the purpose and importance of PCC. This may have influenced their utilization because of these women to utilize the PCC services; they must be knowledgeable about the existing services.

Accordingly single women were 93.8% (AOR: 0.062; 95% CI: 0.007, 0.585) less likely to utilize preconception care than married. This is consistent with studies done in Oklahoma [25]. This might be due to cultural influence in Ethiopia regarding sexual and reproductive life before marriage may make them to fear and not utilize the service. Also single women’s do not want to have a child before marriage, due to that they have not prepared themselves for pregnancy and unplanned pregnancy is common this make them less utilize the service.

Finally, Women who mentioned there is an available unit for preconception care were 14 times more likely to utilize preconception care than women who don’t know the availability of unit for preconception care (AOR: 13.938;95% CI: 3.516,55.251). This might be due to the fact that if women have information about the availability of the services they might be more interested to utilize it. In addition, women who mentioned that there is no available unit for preconception care delivery were 10 times more likely to utilize preconception care than women who don’t know the availability of unit for preconception care (AOR: 10.027;95% CI: 4.331,23.320). This might be due to the delivery of preconception care services with other health care services, according to WHO (2012) recommendation that PCC may delivered by integrating with other health care services [5].

The qualitative result also supplement this finding in fact that there is no coordinated and well organized delivery of preconception care as a service alone, providing this service with other health care services is mandatory to address the services.

However, this study does have its own limitations. First, the study design makes it difficult to determine the direction of causality. There is also a risk of social desirability bias and interviewer bias. In addition, since women’s were asked for the past experience of the service there may be a risk of recall bias.

## Conclusions

The finding of this study showed that women’s utilization of preconception care is low. A woman’s age, marital status, educational status, knowledge about preconception care services and availability of unit for preconception care were statically associated with utilization of preconception care. It indicated that being married; having a high educational level, good knowledge about preconception care services and knowing the availability of unit for preconception care were increased women’s utilization of preconception care. Therefore, establishing preconception care strategies which can address all the components of preconception care and understanding the views of reproductive age women’s and care providers will be essential when designing effective implementation strategies for improving delivery and uptake of preconception care.

## Data Availability

The data that support the findings of this study are available, but some restrictions may apply to the availability of these data as there are some sensitive issues. However, data are available from the corresponding authors upon reasonable request.

## Abbreviations

AIDS: Acquired immune deficiency syndrome
ANC: Ante natal care
AOR: Adjusted odds ratio
BSC: Bachelor of Science
CDC: Center for Disease Control
CI: Confidence interval
HIV: Human immune virus
HTN: Hypertension
KM: Kilo meter
MSC: Master of science
OR: Odds ratio
PCC: Preconception care
PRAMS: Pregnancy Risk Assessment Monitoring System
SDG: Sustainable Development Goal
SPSS: Statistical product and service solutions
SRS: Simple random sampling
STI: Sexually transmitted infections
UN: United Nation
WHO: World Health Organization
